# Genome mining for drug discovery: progress at the front end

**DOI:** 10.1093/jimb/kuab044

**Published:** 2021-07-19

**Authors:** Richard H Baltz

**Affiliations:** CognoGen Biotechnology Consulting, 7757 Uliva Way, Sarasota, FL 34238, USA

**Keywords:** Actinobacteria, Actinomycete, Combinatorial biosynthesis, Genome mining, Myxobacteria, Nonribosomal peptide synthetase, Polyketide synthase, *Streptomyces*, Synthetic biology

## Abstract

Microbial genome mining for drug discovery and development has been accelerating in recent years, driven by technical advancements in genome sequencing, bioinformatics, metabolomics/metabologenomics, and synthetic biology. Microbial genome mining is a multistep process that starts with the sequencing of microbes that encode multiple secondary metabolites and identifying new and novel secondary metabolite biosynthetic gene clusters (BGCs) to pursue. The initial steps in the process are critical for the overall success, and they encompass the most innovative new technologies to revitalize natural product discovery. As microbial genome mining has matured in recent years, unvalidated conjectures about what microbes to pursue, how to identify legitimate secondary metabolite BGCs, and how to sequence DNA to satisfactory levels of completion have been identified. The solutions to correct the misconceptions around these topics are beginning to be implemented.

**Abbreviations used**
A, adenylation domain
ACP, acyl carrier protein
BGC, biosynthetic gene cluster
C, condensation domain
CAT, NRPS elongation module
CATTe, NRPS elongation/termination module
E, epimerase
FAAL, fatty acyl-AMP ligase
MRSA, methicillin-resistant *Staphylococcus aureus*
NRP, nonribosomal peptide
NRPS, nonribosomal peptide synthetase
PCP, peptidyl carrier protein
PK, polyketide
PKS-I, type I polyketide synthetase
T, thiolation (PCP) domain
Te, thioesterase domain

## Introduction

Natural products continue to be important sources for the discovery and development of antibiotics, immune modulators, and antitumor agents for human medicine, and other products for use in animal health and plant crop protection (Baltz, 2019; Demain, 2014; Katz & Baltz, 2016; Newman & Cragg, 2020). The concept of microbial genome mining to revitalize drug discovery emerged in the early 2000s from the observations that newly sequenced actinomycete genomes encode many more secondary metabolite biosynthetic gene clusters (BGCs) than predicted from known secondary metabolomes (Baltz, 2008; Bentley et al., 2002; Ikeda et al., 2003; Ohnishi et al., 2008; Oliynyk et al., 2007). In recent years, the discovery of new and novel natural products has been accelerating by advancements in the microbial genome mining processes (Bachmann et al., 2014; Baltz, 2017a, 2017c, 2019; Foulston, 2019; Kalkreuter et al., 2020; Mitousis et al., 2020; Musiol-Kroll et al., 2019). Genome mining also provides an ever-expanding set of genetic parts and devices for synthetic biology approaches to facilitate combinatorial biosynthesis of novel variants of biologically active secondary metabolites, such as antibiotics (Baltz, 2018, 2019, 2021; Katz et al., 2018; Whitford et al., 2021; Yuzawa et al., 2018). In this perspective, I focus on a few key advancements and opportunities at the front end of the genome mining discovery process and limit my discussions to bacteria, primarily actinomycetes. I also discuss three examples of applying evolutionary relationships within families of BGCs encoded by nonribosomal peptide synthetase (NRPS) and polyketide synthase (PKS-I) mechanisms to facilitate genome mining of structural variants of pharmacologically privileged chemical scaffolds.

## The Genome Mining Process

The genome mining process involves the identification of new and novel secondary metabolite BGCs for product development. The process from genome sequence analysis to product launch can be described as a nine-step value chain (Baltz, 2019) as follows: (i) identify the most promising microbes and sequence their genomes; (ii) identify new and novel secondary metabolite BGCs and their products; (iii) develop fermentation processes to produce large quantities of targeted molecules; (iv) isolate and characterize molecules of interest; (v) determine biological activities of candidate molecules; (vi) optimize the pharmacological properties of promising molecules by medicinal chemistry and combinatorial biosynthesis; (vii) pursue clinical development of promising molecules; (viii) obtain regulatory approval for molecules that meet clinical success criteria; and (ix) market approved compounds. As with any drug discovery process, failure can occur at any step in the value chain. With microbial genome mining, it is critical to optimize the first two steps in the process to enable success in subsequent steps. Importantly, the first two steps present the largest opportunities for transformative innovation in natural product discovery. All subsequent steps in the process have been implemented successfully in the past, but can be further optimized by applying recent advancements in synthetic biology, metabolomics, metabolic engineering, and other technologies. The downstream steps of the genome mining process are beyond the scope of this perspective. Interested readers are referred to recent reviews on other technical advances relevant to bioinformatics, product expression, and strain engineering (Baltz, 2016; Blin et al., 2019; Kang & Kim, 2021; Kautsar et al., 2021; Lee et al., 2020; Merwin et al., 2020; Navarro-Muñoz et al., 2020; Nguyen et al., 2020; Palazzotto et al., 2019; Ren et al., 2020; Schorn et al., 2021; Sharma et al., 2020; Skinnider et al., 2020; Stahlecker et al., 2021; van der Heul et al., 2018; Wang et al., 2019; Whitford et al., 2021; Xu & Wright, 2019).

Examples of mining individual actinomycete genomes have been successful in identifying new and novel secondary metabolite BGCs because they utilized finished-quality genomes (Aigle et al., 2014; Bentley et al., 2002; Challis, 2014; Ikeda et al., 2003; Ikeda et al., 2014; Iftime et al., 2016; Ohnishi et al., 2008; Oliynyk et al., 2007; Thibessard et al., 2015). Finished-quality genomes facilitated the proper assembly of NRPS and PKS-I genes encoding multimodular multienzymes. In spite of these successful examples, most actinomycete genome sequences currently available in public databases are in draft form. Draft-quality genome sequences are sufficient for the correct assembly of many small, nonrepetitive secondary metabolite BGCs (e.g. RiPPs, terpenes, aminoglycosides, and type II polyketides) but not for large BGCs encoding new and novel secondary metabolites biosynthesized by NRPS of PKS-I mechanisms. NRPS and PKS-I BGCs are typically misassembled in draft genomes (Baltz 2017b, 2017c, 2019; Goldstein et al., 2019; Klassen & Currie, 2012). For example, the daptomycin (NRPS) and spinosad (PKS-I) BGCs have been cloned in BAC and cosmid vectors, respectively, and sequenced to finished quality (Miao et al., 2005; Waldron et al., 2001). Two draft genome sequences of highly related *Streptomyces roseosporus* strains, both producers of daptomycin, were sequenced to draft quality by the Broad Institute, and both had NRPS assembly errors. The worst of the two, the commercial daptomycin producer, had one of the three NRPS subunits (*dptA*) correctly assembled, but two partial segments of *dptBC* were located on different contigs, each fused to heterologous NRPS fragments, and *dptD* was located on two different contigs, one of which was fused to a heterologous NRPS sequence (Baltz, 2017b). The spinosad BGC has five contiguous PKS-I genes, encoding SpnABCDE (Waldron et al., 2001). The draft genome sequence of *Saccharopolyspora spinosa*, the spinosad producer, has *spnA* assembled correctly, *spnB* is truncated, *spnC* is missing, *spnD* is split into two fragments on different contigs, one fused to a heterologous NRPS fragment, and the other fused to two heterologous NRPS fragments, and *spnE* is split into two fragments located on two different contigs (Baltz, 2017b). Furthermore, analysis of NRPS, PKS-I, and mixed NRPS/PKS-I annotated BGC sizes from other actinomycetes by antiSMASH indicated that the average actual BGC sizes from finished genomes are greater than twice as large as the predicted average BGC sizes from draft genomes, supporting the interpretation that many of the latter represent fragmented and misassembled “BGCs” (Baltz, 2017b). Since approximately 60% of important natural products or their derivatives for human medicine, animal health, and plant crop protection are of NRPS/PKS-I biosynthetic origins (Table S1), it is important to have finished-quality genomic sequences.

Successful genome mining for drug discovery requires having a robust front-end process of obtaining finished-quality genome sequences from the most gifted microbes that encode multiple secondary metabolite BGCs (Baltz, 2017a); rapid bioinformatic dereplication of known BGCs; avoidance of unvalidated hypothetical BGCs (Baltz 2017c, 2019); and identification of new and novel BGCs, supported by recent advances in metabolomic analyses (Doroghazi et al., 2014; Goering et al., 2016; Schorn et al., 2021). These guiding principles are imbedded in the first two steps in the value chain. Having a robust front-end of the drug discovery process is critical for the development of an overall process that consistently generates new product launches to meet important medical and agricultural needs.

## DNA sequencing technology

The short-read massively parallel DNA sequencing paradigm using Illumina technology has been largely responsible for the rapid accumulation of draft genome assemblies publically available at the National Center for Biotechnology Information (nih.gov) (NCBI). This has been associated with the concept of “high-quality draft genomes” that can be generated very inexpensively. This high-quality notion is adequate for surveying large numbers of genomes for the presence or absence of normal-size genes (e.g. genes involved in primary metabolism or antibiotic resistance). It is not adequate for very large genes encoding repetitive functional domains such as NRPS or PKS-I genes.

Recently, significant advances have been made in long-read DNA sequencing methods developed by Pacific Biosciences (PacBio) and Oxford Nanopore Technologies (MinION). These methods can be coupled with Illumina short-read sequencing to obtain finished-quality sequences of very large genomes from microorganisms gifted for natural product biosynthesis, and which encode multiple BGCs that employ NRPS/PKS-I biosynthetic mechanisms (Gren et al., 2020; Gomez-Escribano et al., 2016; Gomez-Escribano et al., 2021; Gomez-Escribano et al., 2016; Goldstein et al., 2019; Guo et al., 2020; Heinsch et al., 2019; Kono & Arakawa 2019; Lu et al., 2016; Naômé et al., 2018; Nindita et al., 2019; Tian et al., 2015). These advances bode well for obtaining finished-quality genome sequences from many additional gifted microbes.

## Rationale for sequencing gifted microbes that produce known secondary metabolites

One of the important aspects of natural product discovery in the pre-genomic era that led to the demise of previously successful programs in the pharmaceutical industry was the problem of rediscovery of known compounds (Katz & Baltz, 2016). This was a major contributor to diminishing returns on investment that were not sustainable. An important advantage of microbial genome mining is the ability to focus on compounds predicted to be truly novel, and on derivatives of highly active molecules, based initially on bioinformatic analysis alone (Bachmann et al., 2014; Baltz, 2019, 2021). To accomplish this efficiently, it is important to have publicly available sequences from microbes that produce known compounds to serve as an open resource for rapid bioinformatic dereplication of known BGCs. In addition, such a database can serve as a source for mining other new and novel BGCs encoded by the reference genomes, and to support ongoing studies on the evolution and ecology of BGCs that can be applied to synthetic biology and combinatorial biosynthesis approaches to drug discovery (Baltz, 2018, 2021; Chevrette & Currie, 2019; Chevrette et al., 2019; Chevrette et al., 2020; Culp et al., 2020; van Bergeijk et al., 2020; Waglechner et al., 2019).

## Status of sequencing important secondary metabolite producing microbes

The question of which microbes encode the largest numbers of druglike natural products has been addressed recently (Baltz, 2017a, 2019). The most gifted microbes are those with large genomes, including many filamentous actinomycetes (dominated by *Streptomyces* species), and myxobacteria. Others include *Burkholderia, Paenibacillus*, and some species of Cyanobacteria. Those with small genomes, including *Archaea* and many unculturable bacteria, are the least gifted (Baltz, 2017a).

Important questions to address are the following: (i) how many finished genomes from gifted microbes are currently available in public databases to support bioinformatic dereplication and drug discovery by genome mining? and (ii) have we made significant progress in the expansion of finished genomes publically available in recent years?

### Publically available genome sequences

In July of 2020, there were 241,900 bacterial genome sequences available at NCBI (Table 1). Of these, 7.2% were of finished quality. Only 10% of the genome sequences were from Actinobacteria. From a drug discovery perspective, the most important Actinobacteria fall within the filamentous actinomycete genera, including *Streptomyces, Micromonospora, Amycolatopsis, Actinoplanes, Saccharopolyspora*, and others (Baltz, 2017a, 2017c; Katz & Baltz, 2016). Table 1 shows the numbers of filamentous actinomycete genome sequences for 14 genera that produce important secondary metabolites, ranging from 1,864 for *Streptomyces* to 1 for *Streptoalloteichus*, a tobramycin producer. The total filamentous actinomycete genome sequences (2,264) account for 0.98% of total bacterial sequences, whereas the pathogenic *Mycobacterium* sp. alone account for 7,497 of the Actinobacteria sequences. The Gamma Proteobacteria account for 122,764 sequences or 50.7% of the total. These are dominated by human pathogens, such as *Escherichia coli, Pseudomonas* sp., and *Burkholderia* sp. In contrast, within the Delta/Epsilon Proteobacteria, the Myxobacteria comprise only 287 sequences or 0.12% of the total. The Firmicutes account for 62,950 sequences (26% of total), and these are dominated by human pathogens, such as *Staphylococcus* and *Streptococcus* species. Thus, the bacterial genome sequences available in NCBI are heavily skewed in favor of human pathogens (approximately 75%) and underrepresented by genomes from filamentous actinomycetes and myxobacteria (approximately 1.1%), the highly gifted producers of antibiotics used to treat pathogens (Baltz, 2017a). Furthermore, just over 10% of filamentous actinomycete and myxobacterial genomes are of finished quality, so the approximately 90% of draft-quality genomes, mostly assembled by short-read Illumina technology, are not suitable to identify novel secondary metabolite BGCs that employ NRPS or PKS-I biosynthetic mechanisms, because of misassembly issues (Baltz, 2017b, 2018, 2019; Klassen & Currie, 2012, Goldstein et al. 2019). Finishing many existing draft genomes, and increasing the numbers of finished genomes of gifted secondary metabolite producers available in NCBI has the potential to accelerate successful genome mining and combinatorial biosynthesis for drug discovery in the coming years.

**Table 1.** Bacterial Genome Sequences Available at NCBI Genome^a^

Microbial group	Genome assemblies	% of total	Finished assemblies	% Finished
Bacteria	241,900	100	17,449	7.2
Proteobacteria	122,764	50.7	9,880	8.0
Gamma	86,505	35.8	6,354	7.3
*Escherichia coli*	22,420	9.3	1,091	4.9
*Pseudomonas* sp.	10,321	4.3	610	5.9
*Pseudomonas aeruginosa*	5,126	2.1	226	4.4
*Burkholderia* sp.	3,258	1.3	281	8.6
Delta/epsilon	1,960	0.8	106	5.4
Myxobacteria	287	0.12	33	11.5
Firmacutes	62,950	26.0	3,971	6.3
*Staphylococcus*	14,259	5.9	715	5.0
*Streptococcus*	15,594	6.4	695	4.5
Actinobacteria	23,106	9.6	1,715	7.4
*Mycobacterium*	7,497	3.1	229	3.1
*Mycobacterium tuberculosis*	6,719	2.8	195	2.9
*Nocardia*	190	0.08	19	10.0
Filamentous actinomycetes	2,364	0.98	248	10.5
*Streptomyces*	1,864	0.77	210	11.3
*Micromonospora*	161	0.07	7	4.3
*Amycolatopsis*	103	0.04	12	11.7
*Salinispora*	98	0.04	2	2.0
*Nonomuraea*	32	0.01	2	6.3
*Actinoplanes*	28	0.01	8	28.6
*Saccharopolyspora*	25	0.01	2	8.0
*Saccharomonospora*	19	0.008	2	10.5
*Saccharothrix*	14	0.006	2	14.3
*Streptosporangium*	8	0.003	1	12.5
*Lechevalieria*	8	0.003	0	0
*Dactylosporangium*	2	0.0008	0	0
*Planobispora*	1	0.0004	0	0
*Streptoalloteichus*	1	0.0004	0	0

^a^Data assembled in July, 2020.

### Expansion of publically available microbial genome sequences

On August 16, 2016, 71,863 bacterial genome sequences were available in NCBI, of which 7.7% were finished (Baltz, 2017c) (Table 2). Therefore, total bacterial genome sequences have expanded 3.37-fold in about 4 years. *E. coli* genomes expanded 4.76-fold, from 4,706 to 22,420 in that timeframe, whereas myxobacterial sequences expanded from 41 to 287 (7.0-fold), and *Streptomyces* sequences expanded from 752 to 1,864 (2.48-fold). Somewhat discouragingly, the total numbers of finished myxobacterial and *Streptomyces* sequences have only expanded from 19 to 33, and 51 to 210, respectively. This defines a clear opportunity for future improvements in populating public databases with finished-quality sequences from gifted microbes to support drug discovery.

**Table 2.** Increase in Bacterial Genome Sequences from 2016 to 2020

Microbial group	Genome assemblies 2016^a^ (% finished)	% of total 2016^a^	Genome assemblies 2020^b^ (% finished)	% Of total 2020^b^	Genome assembly ratio (2020/2016)
**Bacteria**	71,863 (NR^c^)	100	241,900 (7.2)	100	3.37
**Proteobacteria**	33,627 (8.5)	46.8	122,764 (8.0)	50.7	3.65
*Escherichia coli*	4,706 (4.1)	6.5	22,420 (4.9)	9.3	4.76
*Pseudomonas* sp.	2,625 (6.4)	3.7	10,321 (5.9)	4.3	3.93
*Burkholderia* sp.	1,210 (10.4)	1.7	3,258 (8.6)	1.3	2.69
Myxobacteria	41 (46.3)	0.06	287 (11.5)	0.12	7.00
**Firmacutes**	NR	–	62,950 (6.3)	26.0	–
**Actinobacteria**	7.863 (7.7)	10.9	23,106 (7.4)	9.6	2.94
*Mycobacterium* sp.	4,527 (3.4)	6.3	7,497 (3.1)	3.1	1.66
*Nocardia* sp.	103 (4.9)	0.14	190 (10.0)	0.08	1.84
*Streptomyces*	752 (6.8)	1.05	1,864 (11.3)	0.77	2.48
*Micromonospora*	21 (9.5)	0.03	161 (4.3)	0.07	7.67
*Amycolatopsis*	35 (25.7)	0.05	103 (11.7)	0.04	2.94
*Salinispora*	NR	–	98 (2.0)	0.04	–
*Nonomuraea*	NR	–	32 (6.3)	0.01	–
*Actinoplanes*	12 (33.3)	0.02	28 (28.6)	0.01	2.33
*Saccharopolyspora*	7 (14.3)	0.01	25 (8.0)	0.01	3.57
*Saccharomonospora*	NR	–	19 (10.7)	0.008	–
*Saccharothrix*	NR	–	14 (14.3)	0.006	–
*Streptosporangium*	3 (33.3)	0.004	8 (12.5)	0.003	2.67
*Lechaveleria*	NR	–	8 (0)	0.003	–
*Dactylosporangium*	2 (0)	0.003	2 (0)	0.0008	1.00
*Planobispora*	NR	–	1 (0)	0.0004	–
*Streptoalloteichus*	NR	–	1 (0)	0.0004	–

^a^Data assembled from NCBI in August, 2016 (from Baltz, 2017c).

^b^Data assembled from NCBI in July, 2020.

^c^NR, not reported.

It is important to have finished-quality genome sequences from strains that produce known secondary metabolites to provide a baseline for bioinformatic dereplication to accelerate the discovery of new and novel secondary metabolites (Baltz, 2017c, 2019). They help support the important bioinformatic tools of antiSMASH, *m*inimal *i*nformation about a *bi*osynthetic *g*ene cluster (MIBiG), and IMG-ABC discussed below. As a starting point, the genome sequencing status of actinomycetes that produce 100 important antibiotics and other secondary metabolites was assembled in mid-2016 (Baltz, 2017c). The 100 compounds were produced by 94 actinomycetes, of which 56% had been sequenced, 28% to finished quality. The list was expanded to 142 secondary metabolites in July of 2020, and MIBiG status of the BGCs was included (Table S1). In some cases, more than one producing organism is included in Table S1 for various reasons. Some strains produce more than one of the 142 secondary metabolites (e.g. *S. roseosporus* produces daptomycin, arylomycin, napsamycin, and stenothricin, the latter three identified by genome mining coupled with MS/MS-based networking and peptidogenomics) (Liu et al., 2014). Genome sequences are available for producers of 98 of the compounds (69%), of which 53 have been finished (37.3%) (Table S2). 109 of the BGCs are available in MIBiG (79.6%), although MIBiG entries are not always matched with the original producers, or with producers with finished genomes. Also, 36 of the secondary metabolites in MIBiG are from sequenced BGCs not associated with the draft or finished genome sequences.

Much progress has been made in the last 4 years, but much more work can be done to provide finished genomes for the producers of important secondary metabolites, as exemplified in Table S1, to help provide a more robust open-access dataset for future genome mining efforts. The list of 142 important secondary metabolites produced by actinomycetes is a starting point, and should be expanded to encompass a more complete list of genomes to sequence to finished quality, and include a broader phylogenetic exposure to gifted microbes. It is noteworthy that 58% of the original 100 BGCs and 66.4% of the expanded list of 142 BGCs are of NRPS/PKS-I biosynthetic origins, further emphasizing the need to obtain finished-quality genomes to effectively mine these important BGCs that are routinely misassembled in draft genomes (Baltz 2017b, 2019; Goldstein et al., 2019; Klassen & Currie, 2012).

## Bioinformatic analyses of secondary metabolite BGCs

### antiSMASH

antiSMASH was developed as a stand-alone bioinformatics tool to survey whole genome sequences for the presence of secondary metabolite BGCs. The first version was introduced in 2011 (Medema et al., 2011), followed by five upgrades to the present antiSMASH 6.0 (Blin et al., 2013, 2017, 2019, 2021; Weber et al., 2015). This extraordinary tool is very easy to use and is supported by the antiSMASH database (Blin et al., 2017, 2019, 2020). From a drug discovery perspective, antiSMASH 5.0 is very fast, and it can assign 58 different validated secondary metabolite BGC types. antiSMASH 6.0 has further improved the number of BGC types detected to 71. antiSMASH 3.0 (Weber et al., 2015) incorporated MIBiG (Medema et al., 2015; see below) to improve comparative analyses of assigned BGCs to known BGCs. It also incorporated ClusterFinder (Cimermancic et al., 2014), a feature that provided an optional search of genome sequences for more speculative, unvalidated secondary metabolite BGCs (Baltz, 2019). The speculative nature of ClusterFinder hits (CF_saccharides, CF_fatty acids, and CF_putatives) was not explained well in the initial publications, and many antiSMASH users interpreted all ClusterFinder hits as legitimate BGCs, resulting in erroneous interpretations of the data (Baltz, 2019). The original concept of ClusterFinder was for use as a research tool to help discover totally new types of secondary metabolites, and not as a validated search method for drug discovery. The use of antiSMASH 3.0 with ClusterFinder OFF was a useful method to search finished microbial genomes for validated secondary metabolite BGCs and was used effectively to identify the most gifted microorganisms to pursue drug discovery (Baltz, 2017a, 2017c). antiSMASH analysis of many of the same gifted and nongifted microbes with ClusterFinder ON was also evaluated, and total BGC numbers were inflated by 1.3- to 17-fold, depending on genome size and on whether the microbe was naturally gifted (or not) for secondary metabolite BGC content (Baltz, 2019). The largest discrepancies were observed in microbes with small genomes encoding small numbers of legitimate BGCs, thus giving rise to grossly misleading interpretations on the suitability of certain microbes to serve as robust sources of BGCs for genome mining (Baltz, 2019). Importantly, antiSMASH 5.0 (Blin et al., 2019) removed ClusterFinder from its menu of standard options, so this source of artifactual unvalidated “BGCs” has been mitigated. This serves as a major improvement in the front end of the genome mining process for drug discovery.

### The antiSMASH database

The antiSMASH database is an important adjunct to the antiSMASH bioinformatics tool. Whereas antiSMASH analyzes and annotates individual microbial genomes, the antiSMASH database makes precomputed antiSMASH data from many microbes instantly available for comparative studies. It contains BGCs from 25,236 high-quality bacterial, 388 archaeal, and 177 fungal genomes, and has been incorporated into antiSMASH 5.0 as a ClusterBlast search option. This can be used in conjunction with the KnownClusterBlast option to compare BGCs with manually curated known BGCs from MIBiG 2.0 (Kautsar et al., 2020).

### MIBiG

The MIBiG bioinformatics tool was developed by a collaborative effort of many scientists from around the world to standardize information about relationships between known secondary metabolites and their characterized BGCs (Medema et al., 2015). The authors anticipated that MIBiG would help scientists to connect genes and chemical structures and vice versa, to better understand secondary metabolite biosynthesis, to serve as an evidence-based parts registry for secondary metabolite biosynthetic genes that can be drawn upon for modification of existing biosynthetic pathways and for designing new pathways. From a drug discovery perspective, the MIBiG bioinformatics tool also serves to dereplicate known BGCs and helps identify new and novel BGCs when coupled with antiSMASH. The initial database contained 1,170 BGCs annotated with a minimal number of parameters. Of these, 405 BGCs were more fully annotated by 81 academic groups and some companies worldwide, and the authors anticipated further curation efforts to more fully annotate the other 765 BGCs. MIBiG 2.0 was recently published (Kautsar et al., 2020). Over the span of 5 years, 851 new BGCs have been added to the database, and all entries have undergone extensive re-annotation to increase the overall quality of data. The online repository of BGC data has new functionalities to make the extraction of information more user-friendly. Importantly, MIBiG 2.0 is imbedded in antiSMASH 5.0 and 6.0, and in IMG-ABC 5.0 for rapid whole-genome analyses.

### IMG-ABC

The *i*ntegrated *m*icrobial *g*enomes *a*tlas of *b*iosynthetic gene *c*lusters (IMG-ABC) of the Joint Genome Institute (JGI) of the Department of Energy was developed to couple computational searching of large genomic datasets with the discovery of small molecules (Hadjithomas et al., 2015). At the time, it was the largest repository of information on potential secondary metabolite BGCs and contained about 1000 BGCs that were linked to known structures in the MIBiG database (Medema et al., 2015), and about 900,000 possible BGCs not linked to chemical structures. This massive number of possible BGCs included very large numbers of putative BGCs generated by ClusterFinder analysis using earlier versions of antiSMASH (Hadjithomas et al., 2015, 2017), as well as fragmented NRPS and PKS-I BGCs generated by Illumina sequencing (Baltz, 2017c). The lack of validation of ClusterFinder as a predictor of legitimate secondary metabolite BGCs has been addressed recently (Baltz, 2019). The developers of antiSMASH have recently come to the same conclusions on putative BGCs, and have removed the ClusterFinder option in antiSMASH 5.0 and 6.0. The most recent version of IMG-ABC (v.5.0) has also addressed the problem of unvalidated BGCs generated by ClusterFinder by reanalyzing all genomes by antiSMASH 5.0 and discarding all putative BGCs annotated by earlier versions of antiSMASH with ClusterFinder ON (Palaniappan et al., 2020). The current database contains 1,285 experimentally verified BGCs from MIBiG 2.0 (Kautsar et al., 2020), as well as 317,423 predicted BGCs from 42,892 publically available microbial genomes, and 12,176 predicted BGCs from 4,944 high-quality scaffold bins and other uncultured microbes. These numbers are not artificially inflated by putative BGCs as in the early versions of IMG-ABC (Hadjithomas et al., 2015, 2017), and are more in line with BGC numbers derived by antiSMASH 3.0 with ClusterFinder OFF (Baltz, 2017a, 2017c). This upgrade in IMG-ABC 5.0 is a major advancement in quality that will serve the natural products community and drug discovery efforts well in the coming years.

## Examples of genome mining by exploiting evolutionary relationships and molecular beacons

In order to effectively mine microbial genomes for new and novel natural product BGCs, it is critical that we understand the evolutionary relationships between BGCs, particularly those most likely to have druglike properties. Natural products that have been successfully developed for human medicine, animal health, and plant crop protection are dominated by large molecules biosynthesized by NRPS, PKS-I, and mixed NRPS/PKS-I mechanisms (Katz & Baltz, 2016; Baltz, 2017c, 2019) (Table S1). Since these utilize very large, multimodular, multienzymes, they are subject to evolutionary divergence over millions of years to generate natural combinatorial groupings of somewhat related biosynthetic products. One approach to microbial genome mining is to identify sets of BGCs that are related to known natural products with important clinical applications. For the purposes of this perspective, I review three examples that employ NRPS/PKS-I mechanisms: glycopeptides related to vancomycin, mixed NRP-PKs related to rapamycin and FK506, and lipopeptides related to daptomycin.

## Glycopeptides related to vancomycin

It has been known for decades that antibiotic-producing actinomycetes typically encode self-resistance mechanisms to avoid suicide (Cundliffe & Demain, 2010). The resistance genes typically reside within antibiotic BGCs and can function by a number of mechanisms, including target modification, antibiotic modification, efflux, and duplication of a gene that encodes the antibiotic target, followed by the divergence of one gene copy to encode an antibiotic-resistant target that expresses dominance in the presence of the antibiotic. The gene duplication mechanism can be searched bioinformatically for the presence of second copies of housekeeping genes located in cryptic BGCs. Such BGCs are likely to encode novel mechanisms of action and can be prioritized for antibiotic discovery (Mungen et al., 2020).

Vancomycin is an important glycopeptide antibiotic used to treat infections caused by Gram-positive pathogens, including methicillin-resistant *Staphylococcus aureus* (MRSA). Other clinically approved glycopeptides or lipoglycopeptides related to vancomycin are teicoplanin, oritavancin (a semisynthetic derivative of chloroeremomycin), dalbavancin, and telavancin (David & Daum, 2017; Lampejo, 2020). Many semisynthetic derivatives of glycopeptides have been evaluated over the years (Malabarba et al. 1997). Because of their common mechanisms of action, they all have resistance mechanisms to remodel peptidoglycan to interfere with target binding. Thus, many additional glycopeptide antibiotics have been discovered by selecting soil-dwelling actinomycetes for vancomycin resistance (Thaker et al., 2013, 2014).

Gerry Wright and colleagues have studied the phylogenic relationships between the known glycopeptide antibiotic BGCs, as well as other related glycopeptide BGCs identified by genome mining (Waglechner et al., 2019). The glycopeptides have diverse chemical structures and complex phylogenetic relationships. The core NRPS-derived heptapeptide structure evolved about 300 to 500 million years ago. While many of the 71 BGCs examined contained typical vancomycin-resistance genes (*vanHAX*), others did not. They reasoned that the glycopeptides lacking vancomycin resistance genes may function by different mechanism(s) of action. They showed that the known glycopeptide, complestatin, and the newly discovered corbomycin BGCs are present in clades of five and four, respectively, of the 21 glycopeptide BGCs lacking vancomycin-resistance genes (Culp et al., 2020). They studied the mechanism of action of these potent antibiotics with Gram-positive activities and demonstrated that they inhibit peptidoglycan remodelling during cell division by binding to peptidoglycan and blocking the activities of multiple autolysins. This novel mechanism of action is not subject to single-step mutations to high-level antibiotic resistance. Importantly, these antibiotics are active against multidrug-resistant clinical isolates and present interesting scaffolds for further development.

## Rapamycin/FK506 analogs

Rapamycin (sirolimus) and FK506 (tacrolimus) are structurally related molecules biosynthesized by hybrid NRPS/PKS-I mechanisms. They have potent immunomodulatory activities approved for prophylaxis of organ transplant rejection (Ban et al., 2016; Yoo et al., 2017). Rapamycin and FK506 function mechanistically by facilitating protein–protein interactions between a ubiquitous peptidyl prolyl isomerase, FK506-binding protein (FKBP12), with mTOR and calcineurin, respectively. Both molecules have a constant region for binding FKBP12, and a variable region for binding mTOR or calcineurin. They both incorporate pipecolic acid in the constant region, and their BGCs include genes encoding lysine cyclodeaminase (KCDA) to convert l-lysine to l-pipecolic acid (Ban et al., 2016; Yoo et al., 2017). Scientists at Warp Drive Bio reasoned that this unique mechanism to facilitate protein–protein interactions to inhibit the activities of proteins involved in immune responses might also be programmed to modulate other proteins by chemical variations in the variable region. Such variations might already have evolved in nature, and can also be generated in the laboratory by combinatorial biosynthesis. To search for novel compounds related to rapamycin and FK506, they sequenced approximately 135,000 actinomycete genomes in pools, then deconvoluted those with KCDA genes. They obtained high-quality genome sequences from the strains by a combination of Illumina and PacBio sequencing to assemble the BGCs (Shigdel et al., 2020). Over 40 strains had rapamycin or FK506 BGCs, and other strains encoded seven novel chemical scaffolds related to rapamycin and FK506. WDB002, produced by *Streptomyces malaysiensis*, had a smaller variable region than rapamycin and FK506. It binds to FKBP12 to form a binary complex which then binds to the human centrosomal protein, CEP250, to form a ternary complex. The CEP250 binding site is a flat coiled coil, a type of protein domain previously not considered to be druggable by small molecules. The authors suggest that FKBP12-assisted ternary complex formation represents a promising approach to drug previously undruggable targets. In a recent study on interactions between human proteins and viral proteins expressed during COVID-19 infections, CEP250 was identified as a host protein that interacts with the viral protein Nsp13 (Gordon et al., 2020). This suggests the possibility that WDB002 might have COVID-19 inhibitory activity via CEP250 ternary complex formation (Shigdel et al., 2020).

## Lipopeptides related to daptomycin

Daptomycin (Cubicin) is a cyclic lipopeptide antibiotic produced by *S. roseosporus*. It is approved to treat complicated skin and skin structure infections (SSSI) caused by Gram-positive pathogens, and *S. aureus* bacteremia, including right-sided endocarditis (Eisenstein et al., 2010). The biosynthesis of daptomycin is well understood, and many derivatives have been produced by semisynthesis and combinatorial biosynthesis (Baltz, 2014, 2021). The phylogenetic relationships between diverse lipopeptides have revealed that they employ biosynthetic mechanisms highly suitable for natural evolution, laboratory-based combinatorial biosynthesis, and divergence in the mechanism of action (Baltz, 2021). The latter may be facilitated by the apparent use of ABC transporters as the sole, target-agnostic mechanisms of resistance. Lipopeptide assembly initiates with the attachment of a long-chain fatty acid to the first amino acid by a mechanism that utilizes a fatty acyl-AMP ligase (FAAL), a free-standing acyl carrier protein (ACP), and a specialized NRPS condensation (^III^C) domain (Baltz, 2021). After peptide chain elongation, the nascent lipopeptide is cyclized and released by a thioesterase (Te) domain located on a terminal CAT-CATTe di-modular NRPS. These initiation and termination mechanisms are observed in BGCs encoding daptomycin, taromycin, A54145, friulimicin, laspartomycin, malacidin, and telomycin (Baltz, 2021). The FAAL, ACP, and di-modular NRPS genes have been used as molecular beacons to search for other uncharacterized lipopeptide BGCs in NCBI. A novel lipopeptide BGC was identified in the finished genome of *Streptomyces ambofaciens*, a fragmented BGC related to that of daptomycin was identified in the draft genome of *Streptomyces sedi*, and a fragmented, but likely novel, lipopeptide BGC was identified in the draft genome of *Streptomyces zhaozhouensis* (Baltz, 2021). This study identified three potentially interesting lipopeptide BGCs for further expression, chemical characterization, and biological activity studies. In two of the cases, the identification of likely new lipopeptide BGCs in *S. sedi and S. zhaozhouensis* serve as examples of the use of molecular “beacon” analysis to prioritize strains with draft genome sequences for finishing.

## Perspective

By the middle of the first decade of the 21st Century, it was becoming clear that microbial genome mining could become a major driver to revitalize a moribund natural products discovery and development industry (Baltz, 2008). In the ensuing years, major advances in genome sequencing technology, bioinformatics, metabolomics, and synthetic biology have accelerated the discovery of new and novel secondary metabolite BGCs for drug development, and are providing an unprecedented source of genetic parts and devices for synthetic biology approaches to combinatorial biosynthesis of complex molecules assembled by NRPS, PKS-I, or mixed NRPS/PKS-I pathways (Baltz, 2018; Yuzawa et al., 2018; Iacovelli et al., 2021; Whitford et al., 2021). As with any new technology, advancements are often driven by hypotheses and conjectures that need to be validated or rejected as misconceptions (Baltz, 2019). Unvalidated conjectures can become the anchors that impede progress if they are in fact misconceptions. Three such conjectures have been recently shown to be misconceptions, one persisting over the last two decades, and two generated during the genomic era (Baltz, 2019). The first misconception is that unculturable microbes will provide an abundant new source of natural products for drug discovery. It is now well established that culturable microbes with large genomes are the most “gifted” for natural product drug discovery, and unculturable microbes with small genomes are very poor sources (Baltz, 2017a, 2017c, 2019). The most gifted are the filamentous actinomycetes and myxobacteria. The second misconception from the genomic era is that the ClusterFinder feature of antiSMASH 3.0 identifies potential BGCs for drug discovery. Misinterpretation of this unvalidated conjecture has led to erroneous overcounting of “BGCs” in ungifted microbes and to the accumulation of unnecessary “genomic noise” in the original IMG-ABC database (Baltz, 2017c, 2019). This issue has been addressed by removing the ClusterFinder feature as an option from antiSMASH 5.0 (Blin et al., 2019), and revamping the IMG-ABC database (Palaniappan et al., 2020). These two major advancements will release the drug discovery process from a major misconception that has been impeding progress on mining legitimate secondary metabolite BGCs. The third misconception derived from advances on short read, inexpensive genome sequencing is that “high-quality draft genomes” are sufficient to identify druglike secondary metabolite BGCs. It is now well established that draft genome sequences typically contain misassemblies of NRPS and PKS-I BGCs, the most important ongoing source of important scaffolds for drug discovery. Whereas the numbers of finished genomes of actinomycetes and myxobacteria available at NCBI have increased over the past 4 years, they remain dwarfed by the numbers of draft genomes. DNA sequencing technology has advanced substantially with PacBio and Oxford Nanopore long-read sequencing that complement Illumina short-read sequencing to obtain finished-quality genome sequences from large microbial genomes that encode multiple druglike secondary metabolites biosynthesized by NRPS and PKS-I mechanisms. Building a much larger public database of finished genomes from known producers of important secondary metabolites will be very important to continue improving bioinformatic tools such as MIBig, IMG-ABC, and antiSMASH. Finishing multiple draft genome sequences from other gifted actinomycetes and myxobacteria, perhaps starting with strains encoding potentially novel BGCs identified by molecular beacon analysis, will also augment the drug discovery process.

In summary, microbial genome mining for drug discovery and development is a nine-step process, and optimizing the first two steps is critical to develop a robust process. Identification and correction of the three major misconceptions at the front end of the process will undoubtedly help streamline a productive microbial genome mining process for drug discovery. An immediate opportunity to improve the process is to generate finished-quality genome sequences of gifted microbes that produce known important secondary metabolites and deposit them in publically available databases to facilitate rapid bioinformatic dereplication and discovery of BGCs encoding new and novel compounds derived from NRPS, PKS-I, and other biosynthetic mechanisms. This will enhance the utility of antiSMASH, MIBiG, and IMG-ABC for all academic and industrial discovery activities. In the long term, finished-quality genome sequences from truly gifted microbes for drug discovery should become the norm, not the exception.

## Supplementary Material

kuab044_Supplemental_File
