# Supplementary material for: Genome mining for drug discovery: progress at the front end
Source: J Ind Microbiol Biotechnol. 2021 Jul 19;48(9-10):kuab044. doi: 10.1093/jimb/kuab044 (PMC8788784; doi:10.1093/jimb/kuab044)
Supplement: kuab044_Supplemental_File [file kuab044_supplemental_file.docx]

**Supplementary Material**

**Genome mining for drug discovery: progress at the front end**

**Richard H. Baltz**

**CognoGen Biotechnology Consulting**

**7757 Uliva Way**

**Sarasota, FL 34238**

**E-mail: rbaltz923@gmail.com**

**Tel.: + 1-317-656-7601**

**Table S1** Genome sequence status of actinomycetes that produce important secondary metabolites

| Secondary metabolite | Biosynthetic origin | Major activity/use | Producing actinomycete | NCBI Genome sequence (Mb)^a^ | MIBiG entry | Reference^b^ |
| --- | --- | --- | --- | --- | --- | --- |
| A201A  A201A  A47934  A54145  A54145  Abyssomicin  Acarbose  Actinomycin  Actinorhodin  Adriamycin (doxorubicin)  Albomycin  Amphomycin  Amphotericin B  Antimycin  Antimycin  Apramycin  Arylomycin  Arylomycin  Ascomycin (FK520)  Avermectin  Avilamycin  Bafilomycin  Bafilomycin  Balhimycin  Bialaphos  Bleomycin  Borrelidin  Bottromycin  Bottromycin  Brasilicardin A  Butenyl-spinosyn  Calcimycin (A23187)  Calicheamicin  Candicidin  Caprazamycin  Capreomycin 65188  Carbomycin  CDA  Cephamycin  Chloramphenicol  Chlorobiocin  Chloroeremomycin  Chlortetracycline  Clavulanic acid  Concanamycin A  Coumermycin  Cyphomycin  D-cycloserine  Daptomycin  Daptomycin  Daptomycin-like  Dalbavancin  Daunorubicin  Echinomycin  Enduracidin  Erythromycin  Filipin  Fosfomycin  Fosfomycin  Friulimicin  GE2270  Geldanamycin  Gentamicin  Glycinocin  Griselimycin  Hygromycin A  Josamycin  Kanamycin  Kinamycin F  Kirromycin  Lactacystin  Lankacidin A and C  Lasalocid  Laspartomycin  Lincomycin  Lipstatin  Mannopeptimycin  Micinamycin  Midecamycin  Milbemycin  Mithramycin  Mitomycin C  Moenomycin  Monensin  Napsamycin  Napsamycin  Napsamycin  Narasin  Nargenicin  Nargenicin  Natamycin (pimaricin)  Natamycin  Neomycin  Netropsin  Nigericin  Nikkomycin  Nocardicin A  Nosiheptide  Nystatin  Oligomycin  Oxytetracycline  Pacidamycin  Parvuline  Pladienolide B  Platencin  Platensimycin  Polyoxin (D)  Pristinamycin IA, IIA  Pseudouridimycin  Puromycin  Ramoplanin  Rapamycin  Rebeccamycin  Reveromycin A  Rifamycin  Ristocetin  Salinomycin  Salinosporamide A  Sanglifehrin  Sansanmycin  Sinefungin  Spectinomycin  Spectinomycin  Spinosad (A835438)  Spiramycin  Staurosporine  Staurosporine  Stenothricin  Stenothricin  Streptomycin  Streptothricin  Streptothricin  Streptozotocin  Tacrolimus (FK506)  Tacrolimus (FK506)  Tallysomycin  Taromycin  Tautomycetin  Tautomycin  Teicoplanin  Telomycin  Tetracenomycin  Tetracycline  Thienamycin  Thiocoraline  Thiostrepton  Thiostrepton  Tiacumicin B*  Lipiaramicin*  Tobramycin  Triostin  Tunicamycin  Tylosin  Undecylprodigiosin  Validamycin  Vancomycin  Viomycin  Virginiamycin  WDB002  Zorbamycin | Nucleoside  Nucleoside  NRPS  NRPS  NRPS  PKS I  Glycoside  NRPS  PKS II  PKS II  NRPS-nucleoside  NRPS  PKS I  NRPS-PKS I  NRPS-PKS I  Aminoglycoside  NRPS  NRPS  NRPS-PKS I  PKS I  Glycoside  PKS I  PKS I  NRPS  NRPS  NRPS-PKS I  PKS I  RiPP  RiPP  Diterpine  PKS I  PKS I  PKS I  PKS I  Nucleoside  NRPS  PKS I  NRPS  NRPS  Shikimate  Aminocoumarine  NRPS  PKS II  Other  PKS-I  Aminocoumarin  PKS I  NRPS  NRPS  NRPS  NRPS  NRPS  PKS II  NRPS  NRPS  PKS I  PKS I  Phosphone  Phosphone  NRPS  RiPP  PKS I  Aminoglycoside  NRPS  NRPS  Aminoglycoside  PKS I  Aminoglycoside  PKS II  NRPS-PKS I  NRPS-PKS I  PKS I  PKS I  NRPS  Other  Fatty acyl-lactone  NRPS  PKS I  PKS I  PKS I  PKS II  Quinone  Phosphoglycolipid  PKS I  NRPS-nucleoside  NRPS-nucleoside  NRPS-nucleoside  PKS I  PKS I  PKS I  PKS I  PKS I  Aminoglycoside  NRPS  PKS I  NRPS-nucleoside  NRPS  RiPP  PKS I  PKS I  PKS II  NRPS-nucleoside  NRPS  PKS I  Diterpine  Diterpine  Nucleoside  NRPS-PKS I  NRPS-nucleoside  Nucleoside  NRPS  NRPS-PKS I  Alkaloid  PKS I  PKS I  NRPS  PKS I  NRPS-PKS I  NRPS-PKS I  NRPS-nucleoside  Nucleoside  Aminoglycoside  Aminoglycoside  PKS I  PKS I  Alkaloid  Alkaloid  NRPS  NRPS  Aminoglycoside  Aminoglycoside  Aminoglycoside  Glyco-nitrosourea  NRPS-PKS I  NRPS-PKS I  NRPS-PKS I  NRPS  PKS I  PKS I  NRPS  NRPS  PKS II  PKS II  Other  NRPS  RiPP  RiPP  PKS I  PKS I  Aminoglycoside  NRPS  Nucleoside  PKS I  Other  Glycoside  NRPS  NRPS  NRPS-PKS I  NRPS-PKS I  NRPS-PKS I | Antibacterial  Antibacterial  Antibacterial  Antibacterial  Antibacterial  Antibacterial  Antidiabetic  Antitumor  Research tool  Antitumor  Antibacterial  Antibacterial  Antifungal  Piscicide  Piscicide  Antibacterial  Antibacterial  Antibacterial  Immunomodulator  Anthelmintic  Antibacterial  Antitumor  Antitumor  Antibacterial  Herbicide  Antitumor  Antitumor  Antibacterial  Antibacterial  Immunomodulator  Insecticide  Research tool  Antitumor  Antifungal  Antitubercular  Antitubercular  Antibacterial  Antibacterial  Antibacterial  Antibacterial  Antibacterial  Antibacterial  Antibacterial  Antibacterial  Antiviral  Antibacterial  Antifungal  Antitubercular  Antibacterial  Antibacterial  Antibacterial?  Antibacterial  Antitumor  Antitumor  Antibacterial  Antibacterial  Antifungal  Antibacterial  Antibacterial  Antibacterial  Antibacterial  Antitumor  Antibacterial  Antibacterial  Antibacterial  Antibacterial  Antibacterial  Antibacterial  Antitumor  Antibacterial  Antitumor  Antitumor  Coccidiostat  Antibacterial  Antibacterial  Antiobesity  Antibacterial  Antibacterial  Antibacterial  Anti-parasidic  Antitumor  Antitumor  Antibacterial  Coccidiostat  Antibacterial  Antibacterial  Antibacterial  Coccidiostat  Antibacterial  Antibacterial  Antifungal  Antifungal  Antibacterial  Antitumor  Coccidiostat  Antifungal  Antibacterial  Antibacterial  Antifungal  Research tool  Antibacterial  Antibacterial  Antibacterial  Antitumor  Antibacterial  Antibacterial  Antifungal  Antibacterial  Antibacterial  Antibacterial; RT  Antibacterial  Immunomodulator  Antitumor  Antitumor  Antibacterial  Antibacterial  Coccidiostat  Antitumor  Immunomodulator  Antibacterial  Antifungal; RT  Antibacterial  Antibacterial  Insecticidal  Antibacterial  Antitumor  Antitumor  Antibacterial  Antibacterial  Anti-tubercular  Antibacterial  Antibacterial  Antitumor  Immunomodulator  Immunomodulator  Antitumor  Antibacterial  Antitumor  Antitumor  Antibacterial  Antibacterial  Antitumor  Antibacterial  Antibacterial  Antitumor  Antibacterial  Antibacterial  Antibacterial  Antibacterial  Antibacterial  Antitumor  Antibacterial  Antibacterial  Research tool  Antifungal  Antibacterial  Antibacterial  Antibacterial  Novel target  Antitumor | *Saccharothrix mutabilis* NRRL 3817  *Marinactinospora thermotolerans* DSM 45145  *Streptomyces toyocaensis* NRRL 15009  *Streptomyces fradiae* NRRL 18160  *Streptomyces griseoluteus* ISP-5360  *Verrucosispora maris* AB-18-032  *Actinoplanes* sp. SE50/110  *Streptomyces anulatus* ATCC 11523  *Streptomyces coelicolor* A3(2)  *Streptomyces peucetius* NRRL WC-3868  *Streptomyces griseus* ATCC 700974  *Streptomyces canus* ATCC 12237  *Streptomyces nodosus* ATCC 14899  *Streptomyces albus* J1074  *Streptomyces* sp. S4*  *Streptoalloteichus hindustanus* ATCC 31157  *Streptomyces* sp. Tü 6075  *Streptomyces roseosporus* NRRL 11379  *Streptomyces hygroscopicus* ATCC 14891  *Streptomyces avermitilis* MA-4680  *Streptomyces viridochromogenes* Tü57  *Streptomyces lohii*  *Kitasatospora setae* KM-6054  *Amycolatopsis balhimycina* DSM 5908  *Streptomyces viridochromogenes* DSM 40736  *Streptomyces verticillus* ATCC 15003  *Streptomyces parvulus* Tü 4055  *Streptomycin bottropensis* ATCC 25435  *Streptomyces scabiei* 87.22  *Nocardia terpenica* IFM 0406  *Saccharopoyspora pogona* NRRL 30141  *Streptomyces chartruensis* NRRL 3882  *Micromonospora echinospora* NRRL 15839  *Streptomyces* sp. FR-008  *Streptomyces sp.* MK730-62F2  *Saccharothrix mutabilis* ATCC 23892  *Streptomyces halstedii* NRRL ISP-5068  *Streptomyces coelicolor* A3(2)  *Streptomyces clavuligerus* ATCC 27064  *Streptomyces venezuelae* ATCC 10712  *Streptomyces roseochromogenes* DS 12.976  *Amycolatopsis orientalis* A82846  *Streptomyces aureofaciens* ATCC 10762  *Streptomyces clavuligerus* ATCC 27064  *Streptomyces neyagawaensis* ATCC 27449  *Streptomyces rishiriensis* DSM 40489  *Streptomyces sp.* ISID311  *Streptomyces lavendulae* ATCC 11924  *Streptomyces roseosporus* NRRL 11379  *Streptomyces roseosporus* NRRL 15998^c^  *Streptomyces sedi* DSM 41942^T^  *Nonomuraea* sp. ATCC 39737  *Streptomyces peucetius* NRRL WC-3868  *Streptomyces lasaliensis* NRRL 3382R  *Streptomyces fungicidicus* ATCC 210123  *Saccharopolyspora erythraea* NRRL 2338  *Streptomyces avermitilis* MA-4680  *Streptomyces wedmorensis* NRRL 3426  *Streptomyces fradiae* NRRL 3417  *Actinoplanes friuliensis* DSM 7358  *Planobispora rosea* ATCC 53733  *Streptomyces hygroscopicus* 17997  *Micromonospora echinospora* ATCC 15837  *Streptomyces malaysiensis* DSM 4137  *Streptomyces muensis* DSM 40835  *Streptomyces hygroscopicus* NRRL 2388  *Streptomyces narbonensis* ATCC 19790  *Streptomyces kanamyceticus* ATCC 12853  *Streptomyces murayamaensis*  *Streptomyces collinus* Tü 365  *Streptomyces lactacystinicus* DSM 43136  *Streptomyces griseofuscus* DSM 40191  *Streptomyces lasaliensis* ATCC 35851  *Streptomyces viridochromogenes* ATCC 29814  *Streptomyces lincolnensis* NRRL 2936  *Streptomyces toxitricini* NRRL 15443  *Streptomyces hygroscopicus* NRRL 30439  *Micromonospora griseorubida* A11725  *Streptomyces mycarofaciens* 1748  *Streptomyces bingchenggensis* BCW-1  *Streptomyces argillaceus* ATCC 12956  *Streptomyces lavendulae* NRRL 2564  *Streptomyces viridosporus* ATCC 14672  *Streptomyces cinnamonensis* ATCC 15413  *Streptomyces* sp. DSM 5940  *Streptomyces sp.* Tu6575  *Streptommyces roseosporus* NRRL 15998  *Streptomyces aureofaciens* NRRL 5758  *Nocardia argintinensis*  *Nocardia* sp. CS682  *Streptomyces natalensis* ATCC 27448  *Streptomyces chattanoogensis* ATCC 13358  *Streptomyces fradiae* ATCC 10745  *Streptomyces ambofaciens* ATCC 23877  *Streptomyces* sp. DSM 4173  *Streptomyces tendae* Tü901  *Nocardia uniformis* ATCC 21806  *Streptomyces actuosus* ATCC 25421  *Streptomyces noursei* ATCC 11455  *Streptomyces avermitilis* MA-4680  *Streptomyces rimosus* ATCC 10970  *Streptomyces coerruleorubidus*  *Streptomyces parvulus* 2297  *Streptomyces platensis* Mer-11107  *Streptomyces platensis* BS12029  *Streptomyces platensis* BS12029  *Streptomyces cacaoi* AS4.1602  *Streptomyces pristinaespiralis* ATCC 25486  *Streptomyces* sp. ID38640  *Streptomyces alboniger* NRRL B-1832  *Actinoplanes* sp. ATCC 33076  *Streptomyces rapamycinicus* NRRL 5491  *Lechevalieria aerocolonegenes* ATCC 39243  *Streptomyces* sp. SN-593  *Amycolatopsis mediterranei* S699  *Amycolatopsis lurida* NRRL 2430  *Streptomyces albus* DSM 41389  *Salinispora tropica* CNB-440  *Streptomyces flaveolus* DSM 9954  *Streptomyces* sp. SS  *Streptomyces griseolus* NRRL B-2925  *Streptomyces spectabilis* ATCC 27465*  *Streptomyces netropsis*  *Saccharopolyspora spinosa* NRRL 18395  *Streptomyces ambofaciens* ATCC 23877  *Streptomyces staurosporeus (Lentzia albida)*  *Streptomyces* sp. TP-A0274  *Streptomyces roseosporus* NRRL 15998  *Streptomyces* sp. Tu 6075  *Streptomyces griseus* NBRC 13350  *Streptomyces lavendulae* NRRL B-2774  *Streptomyces rochei* F20  *Streptomyces acromogenes* NRRL 3125  *Streptomyces tsukubaensis* NRRL 18488  *Streptomyces* sp. MA6548  *Streptoalloteichus hindustanus* ATCC 31158  *Saccharomonospora* sp. CNQ490  *Streptomyces* sp. CK4412  *Streptomyces spiroverticillatus*  *Actinoplanes teichomyceticus* ATCC 31121  *Streptomyces canus* ATCC 12646  *Streptomyces glaucescens* DSM 42922^c^  *Streptomyces rimosus* (multiple)  *Streptomyces cattleya* NRRL 8057  *Micromonospora* sp. ML1  *Streptomyces azureus* ATCC 14921  *Streptomyces laurentii* ATCC 31255  *Dactylosporangium aurantiacum* NRRL 18085  *Dactylosporangium aurantiacum* NRRL B8018  *Streptoalloteicus hindustanus* ATCC 31157  *Streptomyces triostinicus* ATCC 21043  *Streptomyces chartreusis* NRRL 12338  *Streptomyces fradiae* ATCC 19609  *Streptomyces coelicolor* A3(2)  *Streptomyces hygroscopicus jinggangensis* 5008  *Amycolatopsis orientalis* B-37  *Streptomyces vinaceus* ATCC 27476  *Streptomyces virginiae* NRRL-ISP 5094  *Streptomyces malasiensis* DSM 41697^T^  *Streptomyces flavoviridis* ATCC 21892 | --  5.66  7.34  -  6.84  6.73 (F)  9.24 (F)  8.85  9.05 (F)  9.53  -  11.57  7.71 (F)  6.84 (F)  7.61  7.28  7.93 (F)  7.85  -  9.03 (F)  9.69  -  8.76 (F)  10.80  8.65  -  -  8.96  10.15 (F)  9.28  9.44 (F)  8.98 (F)  -  7.26 (F)  -  -  7.74  9.05 (F)  8.54 (F)  8.23 (F)  9.78  -  -  8.54 (F)  9.13  9.12  8.19  -  7.85  7.82  6.60  -  9.53  -  -  8.08 (F)  9.03 (F)  9.38  -  9.38 (F)  8.69  -  7.7  10.74 (F)  -  -  -  10.13 (F)  -  8.27 (F)  -  9.12 (F)  -  10.74 (F)  10.32 (F)  -  -  -  -  11.9 (F)  -  -  8.51  -  -  7.93 (F)  7.82  -  -  8.92  8.65  9.13  6.99 (F)  8.39 (F)  -  -  8.77  8.15 (F)  9.82 (F)  9.03 (F)  9.36 (F)  8.48  8.35  -  -  -  -  8.53 (F)  8.79  7.55  -  12.70  10.64  -  10.24 (F)  8.99 (F)  8.38 (F)  5.18 (F)  10.35  8.12  7.54  9.81 (F)  6.88  8.58  8.27 (F)  9.44  -  7.82  7.93 (F)  8.55 (F)  8.47  -  -  8.02 (F)  -  7.28  4.94  -  -  8.29 (F)  11.57  7.45 (F)  9.36 (F)  8.09 (F)  -  8.79  8.03 (F)  -  11.45  7.28  -  9.91 (F)  7.67  9.05 (F)  10.38 (F)  9.49 (F)  7.67 (F)  8.32  -  - | -  +  +  +  -  +  +  +  +  -  -  -  +  -  +  +  -  +  -  +  +  +  -  +  -  +  +  +  +  +  -  +  +  +  -  +  -  +  +  +  -  -  -  +  -  +  +  +  +  -  -  -  +  +  +  +  +  -  +  -  +  +  +  -  +  -  -  +  +  +  -  -  +  +  +  +  +  -  +  -  +  +  +  +  +  -  -  -  +  -  -  +  +  +  +  -  +  +  +  +  +  +  -  + + +  + -  +  +  -  +  +  - + + + +  +  -  -  -  +  +  +  -  +  +  -  +  -  +  -  - + + + + + + +  +  +  + +  -  +  +  -  +  +  +  -  + + + +  +  -  + | Sauger et al., 2017  N, M  N, M  M  Baltz, 2018; Baltz, 2019  N, M  N, M  N, M  N, M  N, Lomovskaya et al., 1999  Zeng et al., 2012  N, Baltz, 2021  N, M  N, Olano et al., 2014  N, M  N, M  N  N, M  Andexer et al., 2011  N, M  N, M  M  N, Nara et al., 2017  N, M  N, Raibaud et al., 1991  M  M  N, M  N, M  N, M  N, Hahn et al., 2006  N, M  M  N, M  Kaysser et al., 2009  M  N. -  N, M  N, M  N, M  N, Pojer et al., 2002  van Wageningen et al., 1998  Wang et al., 2019  N, M  Haydock et al., 2005  N, M  N, M  M  N, M  N, McHenney & Baltz, 1996  N, Baltz, 2021  Sosio et al., 2003  N, M  M  M  N, M  N, M  N, Ju et al., 2015  M  N, Müller, et al., 2007  N, M  M  N, M  N, Baltz, 2021  M  Palaniappan et al., 2006  Arsic et al., 2018  N, M  M  N, M  Také et al., 2015  N, Gren et al., 2021  M  N, M  N, M  M  M  Anzai et al., 2003  M  N, Zhang et al., 2016  M  M  N, M  M  M  N  N, Liu et al., 2014  Dorman et al., 1976  M  N, Dhakal et al., 2020  N, Mendes et al., 2007  N, M  N, M  N, M  M  Lauer et al., 2000  N, M  N, M  N, M  N, M  N, M  N, M  N, Baltz, 2021  M  M  M  M  N, Mast et al., 2010  N, M  N, M  Hoertz et al., 2012  N, M  N, M  Miyazawa et al., 2015  N, M  N, M  N, M  N, M  N, M  N, Li et al., 2013  N, Oshima et al., 2015  N, Kim et al., 2008  N, M  N, M  N, M  N, Onaka et al., 2002  M  N, M  N  N, M  N  M  Ng et al., 2019  N, Ban et al., 2016  M  N, M  N, M  M  M  N, M  N, M  N, M  N, M  N, M  M  M  N, M  M  N  N, M  M  N, M  N, Cundliffe, 2008  N, M  N, M  N, M  N, M  N, M  Shigdel et al., 2020  M |

Abbreviations: PKS I, type I polyketide synthase; PKS II, type II PKS; NRPS, nonribosomal peptide synthetase; NRPS-PKS I, mixed pathway; RiPP, ribosomally synthesized and post-translationally modified peptide.

^a^ (F), finished sequence; -, not sequenced. All others have draft sequences.

^b^ References to genome sequences in NCBI (N) and BGCs in MIBiG 2.0 (M) [Kautzer et al., 2020] can be found at <https://www.ncbi.nlm.nih.gov/genome/> and <https://mibig.secondarymetabolites.org/>, respectively.

^c^ Daptomycin high producer is the same as strain A21978.65 (McHenney and Baltz, 1996).

**Table S2** Summary of DNA sequencing status of actinomycetes that produce 142 important secondary metabolites

| Sequencing status of producers | BGC producer (% of total) | BGC in MIBiG 2.0 (% of total) |
| --- | --- | --- |
| Finished  Draft  Not sequenced  Total | 53 (37.3)  45 (31.7)  44 (31.0)  142 (100) | 40 (28.2)  33 (23.2)  36 (25.4)  109 (76.8) |

**References**

Andexer, J. N., Kendrew, S. G., Nur-e-Alam, M. et al. (2011). Biosynthesis of the immunosuppressants FK506, FK520, and rapamycin involves a previously undescribed family of enzymes acting on chorismite. *Proceedings of the National Academy of Sciences of the United States of America*, 108, 4776-4781.

Anzai, Y, Saito, N., Tanaka, M., Kinoshita, K., Koyama, Y, & Kato, F. (2003). Organization of the biosynthetic gene cluster for the polyketide macrolide mycinamicin in *Micromonospora griseorubida*. *FEMS Microbiology Letters*, 218, 1335-141.

Baltz, R. H. (2018). Synthetic biology, genome mining, and combinatorial biosynthesis of NRPS-derived antibiotics: a perspective. *Journal of Industrial Microbiology and Biotechnology,* 45, 635-649.

Baltz, R. H. (2019). Natural product drug discovery in the genomic era: realities, conjectures, misconceptions, and opportunities. *Journal of Industrial Microbiology and Biotechnology,* 46, 281-299.

Baltz, R. H. (2021). Genome mining for drug discovery: cyclic lipopeptides related to daptomycin. *Journal of Industrial Microbiology and Biotechnology,* DOI: 10.1093/jimb/kuab020.

Ban, Y. H., Park, S. R., & Yoon, Y. J. (2016). The biosynthetic pathway for FK506 and its engineering: from past achievements to future prospects. *Journal of Industrial Microbiology and Biotechnology,* 43, 389-400.

[Cundliffe](https://pubmed.ncbi.nlm.nih.gov/?sort=date&term=Cundliffe+E&cauthor_id=18852501), E. (2008).  Control of tylosin biosynthesis in *Streptomyces fradiae*. *Journal of Microbiology and Biotechnology,* 18, 1485-1491.

Dhakai, D., Han, J. M., Mishra, R. et al (2020). Characterization of the tailoring step of nargenicin A1 biosynthesis reveals a novel analogue with anticancer activities. *ACS Chemical Biology,* 15, 1370-1380.

Dorman, D. E., Paschal, J. W., Nakatsukasa, W. M., Huckstep, L. L., & Neuss, N. (1976) The use of 13C-NMR. Spectroscopy in biosynthetic studies, II. Biosynthesis of narasin, a new polyether ionophore from fermentation of *Streptomyces aureofaciens*. *Helvetica Chimica Acta,* 59, 2625-2634.

Gren, T., Jergensen, T. S., Whitford, C. M., & Weber, T. (2020). High-quality sequencing, assembly, and annotation of the *Streptomyces griseofuscus* DSM 40191 genome. *Microbiology Resource Announcement* 9, e01100-20.

Hahn, D. R., Gustafson, G., Waldron, C., Bullard, B., Jackson, J. D., Mitchell, J. (2006). Butenyl-spinosyns, a natural example of genetic engineering of antibiotic biosynthetic genes. *Journal of Industrial Microbiology and Biotechnology*, 33, 94-104.

Haydock, S. F., Appleyard, A. N., Mironenko, T., Lester, J., Scott, N., & Leadlay, P. F. (2005). Organization of the biosynthetic gene cluster for the macrolide concanamycin A in *Streptomyces neyagawaensis* ATCC 27449. *Microbiology,* 151, 3161-3169.

Hoertz, A. J., Hamburger, J. B., Goodman, D. M., Bednar, M. M., & McCafferty, D. G. (2012). Studies on the biosynthesis of the lipodepsipeptide ramoplanin A2. *Bioorganic and Medicinal Chemistry*, 20, 859-865.

[Ju](https://pubmed.ncbi.nlm.nih.gov/?sort=date&term=Ju+KS&cauthor_id=26324907), K. S., [Gao](https://pubmed.ncbi.nlm.nih.gov/?sort=date&term=Gao+J&cauthor_id=26324907) J., [Doroghazi](https://pubmed.ncbi.nlm.nih.gov/?sort=date&term=Doroghazi+JR&cauthor_id=26324907), J. R., [Wang](https://pubmed.ncbi.nlm.nih.gov/?sort=date&term=Wang+KK&cauthor_id=26324907), K. K. A., [Thibodeaux](https://pubmed.ncbi.nlm.nih.gov/?sort=date&term=Thibodeaux+CJ&cauthor_id=26324907), C. J., [Steven Li](https://pubmed.ncbi.nlm.nih.gov/?sort=date&term=Li+S&cauthor_id=26324907), S., [Metzger](https://pubmed.ncbi.nlm.nih.gov/?sort=date&term=Metzger+E&cauthor_id=26324907), E., [Fudala](https://pubmed.ncbi.nlm.nih.gov/?sort=date&term=Fudala+J&cauthor_id=26324907), J., [Su](https://pubmed.ncbi.nlm.nih.gov/?sort=date&term=Su+J&cauthor_id=26324907), J., [Zhang](https://pubmed.ncbi.nlm.nih.gov/?sort=date&term=Zhang+JK&cauthor_id=26324907), J. K., [Jaeheon Lee](https://pubmed.ncbi.nlm.nih.gov/?sort=date&term=Lee+J&cauthor_id=26324907), J., [Cioni](https://pubmed.ncbi.nlm.nih.gov/?sort=date&term=Cioni+JP&cauthor_id=26324907), J. P., [Evans](https://pubmed.ncbi.nlm.nih.gov/?sort=date&term=Evans+BS&cauthor_id=26324907), B. S., [Hirota](https://pubmed.ncbi.nlm.nih.gov/?sort=date&term=Hirota+R&cauthor_id=26324907), R., [David P Labeda](https://pubmed.ncbi.nlm.nih.gov/?sort=date&term=Labeda+DP&cauthor_id=26324907), D. P., [van der Donk](https://pubmed.ncbi.nlm.nih.gov/?sort=date&term=van+der+Donk+WA&cauthor_id=26324907), W. A., & [Metcalf](https://pubmed.ncbi.nlm.nih.gov/?sort=date&term=Metcalf+WW&cauthor_id=26324907), W. W. (2015). Discovery of phosphonic acid natural products by mining the genomes of 10,000 actinomycetes. *Proceedings of the National Academy of Sciences of the United States of America*, 112, 12175-12180.

Kaysser, L., Lutsch, L., Siebenberg, S., Wemakor, E., Kammerer, B., & Gust, B. (2009). Identification and manipulation of the caprazamycin gene cluster lead to new simplified liponucleoside antibiotics and give insights into the biosynthetic pathway. *The Journal of Biological Chemistry*, 284, 14987-14996.

Kim, K. R., Kim, T. J., & Suh, J. W. (2008). The gene cluster for spectinomycin biosynthesis and the aminoglycoside-resistance function of *spcM* in *Streptomyces spectabilis*. *Current Microbiology,* 57, 371-374.

Lauer, B., Süssmuth, R., Kaiser D., Jung, G. & Borman, C. (2000). A putative enolpyruvyl transferase gene involved in nikkomycin biosynthesis. *The Journal of Antibiotics*, 53, 385-392.

Li, Q., Wang, L., Xie, Y., Wang, S., Chen, R., & Hong, B. (2013). SsaA, a member of a novel class of transcriptional regulators, controls sansanmycin production on *Streptomyces* sp. strain SS through a feedback mechanism. *Journal of Bacteriology,* 195, 2232-2243.

Mast, Y., Weber, T., Golz, M., Ort-Winklbauer, R., Gondran, A., Wohlleben, W., & Schinko, E. (2010). Characterization of the ‘pristinamycin supercluster’ of *Streptomyces pristinaespiralis*. *Microbial Biotechnology,* 4, 192-206.

Liu, W. T., Lamsa, A., Wong, W. R., Boudreau, P. D., Kersten, R., Peng, Y., Moree, W. J., Duggan, B. M., Moore, B. S., Gerwick, W. H., Linington, R. G., Pogliano, K., & Dorrestein, P. C. (2014). MS/MS-based networking and peptidgenomics guided genome mining revealed the stenothricin gene cluster in *Streptomyces roseosporus*. *Journal of Antibiotics,* 67, 99-104.

Lomovskaya, N., Otten, S. L., Doi-Katayama, Y., Fonstein, L., Liu, X. C., Takatsu, T., Inventi-Solari, A., Fillipini, S., Torti, T., Colombo, A. L., & Hutchinson, C. R. (1999). Doxorubicin overproduction in *Streptomyces peucetius*: cloning and characterization of the *dnrU* ketoreductase and *dnrV* genes and the *doxA* cytochrome P-450 hydroxylase gene. *Journal of Bacteriology*, 181, 305-318.

McHenney, M. A. & Baltz, R. H. (1996). Gene transfer and transposition mutagenesis in *Streptomyces roseosporus*: mapping of insertions that influence daptomycin of pigment production. *Microbiology (Reading, England)*, 142, 2363-2373.

Miyazawa, T., Takahashi, S., Kawata, A., Panthee, S., Hayashi, T., Shimizu, T., Nogawa, T., & Osada, H. (2015). Identification of middle chain fatty acyl-CoA ligase responsible for the biosynthesis of 2-alkylmalonyl-CoAs for polyketide extender unit. *Journal of Biological Chemistry,* 290, 26994-27011.

Müller, C., Nolden, S., Gebhardt, P., Heinzelmann, E., Lange, C., Puk, O., Welzel, K.,Wohlleben,W., & Schwartz, D. (2007). Sequencing andanalysis of the biosynthetic gene cluster of the lipopeptide antibiotic friulimicin in *Actinoplanes friuliensis*. *Antimicrobial Agents and* *Chemotherapy*, 51, 1028–1037.

Nara, A., Hashimoto, T., Komatsu, M., Nishiyama, M., Kuzuyama, T., & Ikeda, H (2017) Characterization of bafilomycin biosynthesis in *Kitasatospora setae* KM-6054 and comparative analysis of gene clusters in *Actinomycetales* microorganisms. *The Journal of Antibiotics,* 70, 616-624.

Ng, T. L., Rohac, R., Mitchell, A, Boal, A. K., & Balskus, E. (2019). An *N*-nitrosating metalloenzyme constructs the pharmacophore of streptozotocin. *Nature*, 566, 94-99.

Olano, C., Garcia, I., González, A. et al. (2014). Activation and identification of five clusters for secondary metabolites in *Streptomyces albus* J1074. *Microbial Biotechnology,* 7, 242-256.

Onaka, H., Taniguchi, S., Igarashi, Y., Furumai, T. (2002). Cloning of the staurosporine biosynthetic gene cluster from *Streptomyces* sp. TP-A0274 and its heterologous expression in *Streptomyces lividans*. *The* *Journal of Antibiotics,* 55,1063-1071.

Oshima, K. Hattori, M., Shimizu, H., Fukuda, K., Nemoto, M., Inagaki, K., & Tamura, T. (2015). Draft genome of *Streptomyces incarnatus* NRRL8089, which produces the nucleoside antibiotic sinefungin. *Genome Announcements,* 3, e00715-15.

Palianiappan, N., Ayers, S., Gupta, S., Habib, E. S., Reynolds, K. A. (2006). Production of hygromycin A analogs in *Streptomyces hygroscopicus* NRRL 2388 through identification and manipulation of the biosynthetic gene cluster. *Chemistry and Biology*, 13, 753-764.

Pojer, F., Li, S. M., & Heide, L. (2002). Molecular cloning and sequence analysis of the clorobiocin biosynthetic gene cluster of aminocoumarin antibiotics. *Microbiology (Reading, England)*, 148, 3901-3911.

Raibaud, A., Zalacain, M., Holt, T. G., Tizard, R., & Thompson, C. J. (1991). Nucleotide sequence analysis reveals linked N-acetyl-hydroxylase, thioesterase, transport, and regulatory genes encoded by the bialophos biosynthetic gene cluster. *Journal of Bacteriology*, 173, 4454-4463.

Sauger, I., Molloy, B., Sanz, E., Sánchez, M. B., Fernández-Lobato, M., & Jiminéz, A. (2017). Characterization of the biosynthetic gene cluster (*ata*) for the A201A aminonucleoside antibiotic from *Saccharothrix mutabilis* subsp. *capreolus.* *The Journal of Antibiotics,* 70, 404-413.

Shigdel, U. K., Lee, S. J., Sowa, M. E. Bowman, B. R., Robison, K., Zhou, M., , K. H., Stiles, D. T., Blodgett, J. A. V., Udwary, D. W. Rajczewski, A. T., Mann, A. S., Mostafavi, S., Hardy, T., Arya, S., Weng, Z., Stewart, M., Kenyon, K., Morgenstern, J. P., Pan, E., Gray, D. C., Pollock, R. M., Fry, A. M., Klausner, R. D., Townson, S. A., & Verdine, G. L. (2020). Genomic discovery of an evolutionarily programmed modality for small-molecule targeting of an intractable protein surface. *Proceedings of the National Academy of Sciences of the United States of America,* 117, 17195-17203.

Sosio, M., Stinchi, S., Beltrametti, F., Lazzarini, A, & Donadio, S. (2003). The gene cluster for the biosynthesis of the glycopeptide antibiotic A40926 by *Nonomuraea* species. *Chemistry and Biology*, 10, 541-549.

Také, A., Matsumoto, A., Ōmura, S., & Takahashi, Y. (2015). *Streptomyces lactacystinicus* sp. nov. and *Streptomyces cyslabdanicus* sp. nov., producing lactacystin and cyslabdan, respectively. *The Journal of Antibiotics,* 68, 322-327.

van Wageningen, A. M., Kirkpatrick, P. N., Williams, D. H., Harris, B. R., Kershaw, J. K., Lennard, N. J., Jones, M., Jones, S. J., & Solenberg, P. J. (1998). Sequencing and analysis of genes involved in the biosynthesis of a vancomycin group antibiotic. *Chemistry and Biology*, 5, 155-162.

Wang, X., Yin, S., Bai, J., Liu, Y., Fan, K, Wang, H., Yuan, F., Zhao, B., Li, Z, & Wang, W. (2019). Heterologous production of chlorotetracycline in an industrial grade *Streptomyces rimosus* host. *Applied Microbiology and Biotechnology*, 103, 6645-6655.

Zeng, Y., Kulkarni, A., Yang, Z., Patil, P. B., Zhou, W. Chi, W., Van Lanen, S., & Chen, S. (2012). Biosynthesis of albomycin δ(2) provides a template for assembling siderophore and aminoacyl-tRNA synthetase inhibitor conjugates. *ACS Chemical Biology* 7, 1565-1575.

Zhang, Y, He, H., Liu, H., Wang, H., Wang, X, & Xiang, H. (2016). Characterization of a pathway-specific activator of milbemycin biosynthesis and improved milbemycin production by its overexpression in *Streptomyces bingchenggensis*. *Microbial Cell Factories*, 15, 152.
